# Supplementary material for: Preclinical Immunogenicity and Efficacy Studies for Therapeutic Vaccines for Human Papillomavirus-Type-16-Associated Cancer
Source: Vaccines (Basel). 2024 Jun 4;12(6):616. doi: 10.3390/vaccines12060616 (PMC11209626; doi:10.3390/vaccines12060616)
Supplement: Supplementary file 1 [file vaccines-12-00616-s001.zip › vaccines-2995823-supplementary.pdf]

Figure S1 for Figures 1 and 6.

Predicted protein sizes

Insert	Characteristics	MW
gDE7652	full-length	81.3kDa
gDE7652	N-terminal degradation product	56.4 kDa
gDE7652	C-terminal degradation product	24.9 kDa
gDHis-tagE7652	full-length	83.8 kDa
gDHis-tagE7652	N-terminal degradation product	58 kDa
gDHis-tagE7652	C-terminal degradation product	25.1 kDA
gDE765	full-length	88.1 kDa

The different inserts, some of their features and their calculated molecular weight are shown.

Figure S2 for Figures 2, 4 and 5

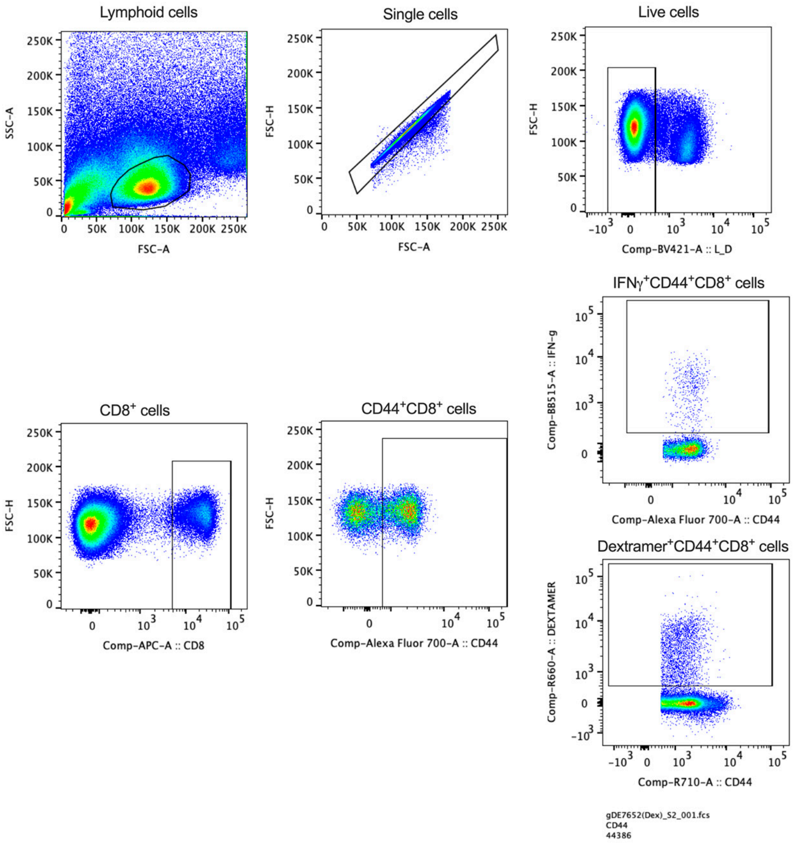

Gating strategy to determine frequencies of CD44⁺CD8⁺ T cells producing IFN-γ or expressing the TCR specific for the immunodominant epitope of E7. Cells were gated first on lymphoid cells, then single cells, then live cells. Live cells were gated on CD8⁺ cells and this subset was gated on those positive for CD44. CD44⁺ cells were gated on cells that stained for intracellular IFN–γ or in a different experiment with the E7-specific dextramer.

Figure S3 relates to Figures 2, 4 and 5

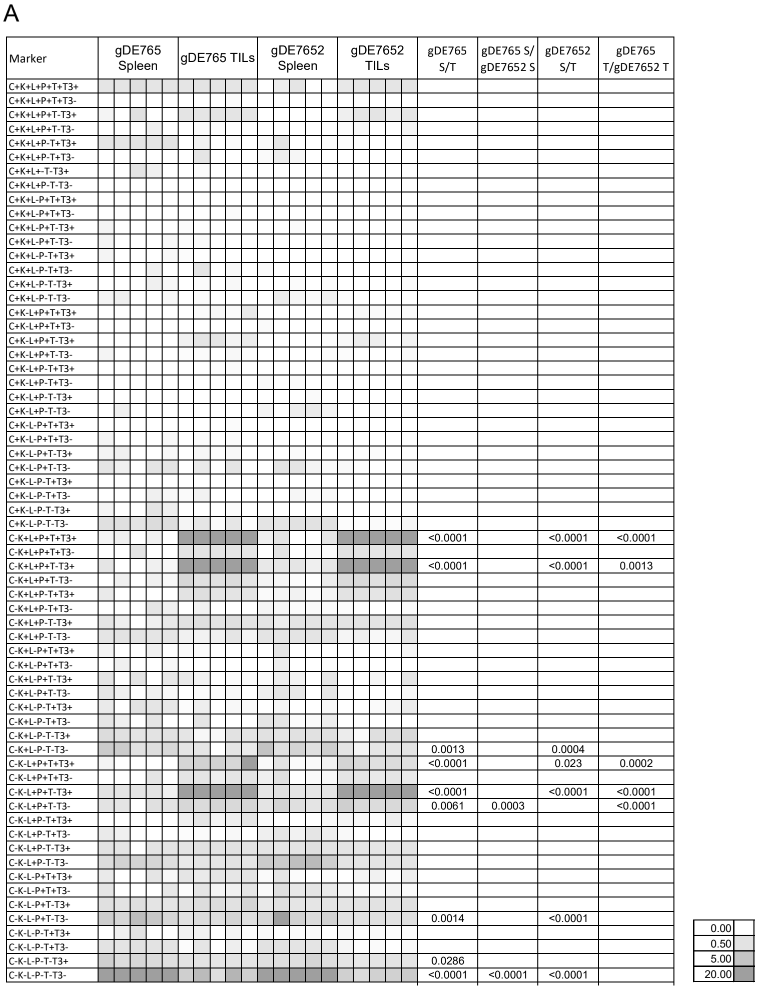

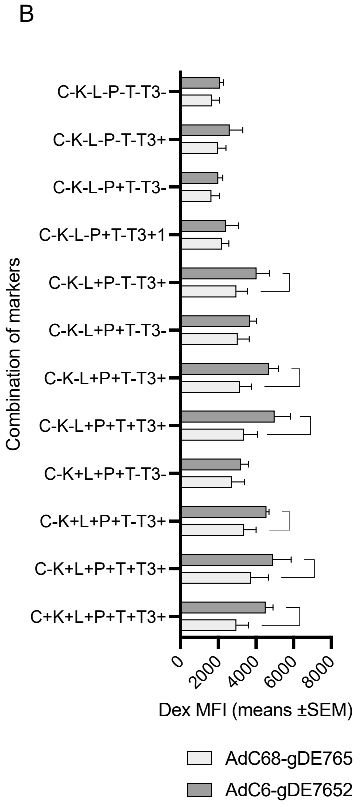

Figure S3.
[A] Shows as a heatmap percentages of E7-dextramer⁺CD8⁺ T cells from spleens and tumors expressing the different combinations of markers. The p-values of significant differences by 2-way Anova are shown to the right. [B] MFI of the E7-dextramer stain of CD8⁺ T cells from spleens and tumors expressing the indicated combinations of markers. Differences were calculated by 2-way Anova. S – T cells from spleens, T – T cells from tumors.

Figure S4 relates to Figure 5

No of markers	AdC6-E7652, d21, spleen	AdC6-E7652, d21, tumors	AdC6-gDE7652, d21, spleen	AdC6 gDE7652, d21, tumor	gDE765 S/T	gDE765 S/ gDE7652 S	gDE7652 S/T	gDE765 T/gDE7652 T	AdC6-E7652, d33, spleen	AdC6-E7652, d33, tumor	AdC6-gDE7652, d 39, spleen	AdC6-gDE7652, d 39, tumor	gDE765 S/T	gDE765 S/ gDE7652 S	gDE7652 S/T	gDE765 T/gDE7652 T	AdC6-E7652 S d21/33	AdC6-E7652 T d21/33	AdC6-gD E7652 S d21/39	AdC6-gD E7652 T d21/39
0.00					<0.0001		<0.0001	<0.0001					<0.0001		<0.0001	<0.0001	<0.0001	<0.0001	<0.0001	<0.0001
1.00								0.0168					<0.0001		0.0006			0.0003	<0.0001	
2.00					<0.0001		0.002											<0.0001		
3.00							<0.0001	<0.0001					<0.0001		<0.0001	<0.0001		<0.0001		<0.0001
4.00													<0.0001		<0.0001	0.0024		<0.0001		0.005
5.00																				
6.00																				

0.00	
0.50	
5.00	
20.00	

Figure S4.

Shows as a heatmap the frequencies of E7 dextramer+ CD8+ T cells expressing 0-6 of the tested differentiation/exhaustion markers. The p-values of significant differences by 2-way Anova are shown to the right. S – T cells from spleens, T – T cells from tumors.

Figure S5 relates to Figure 5

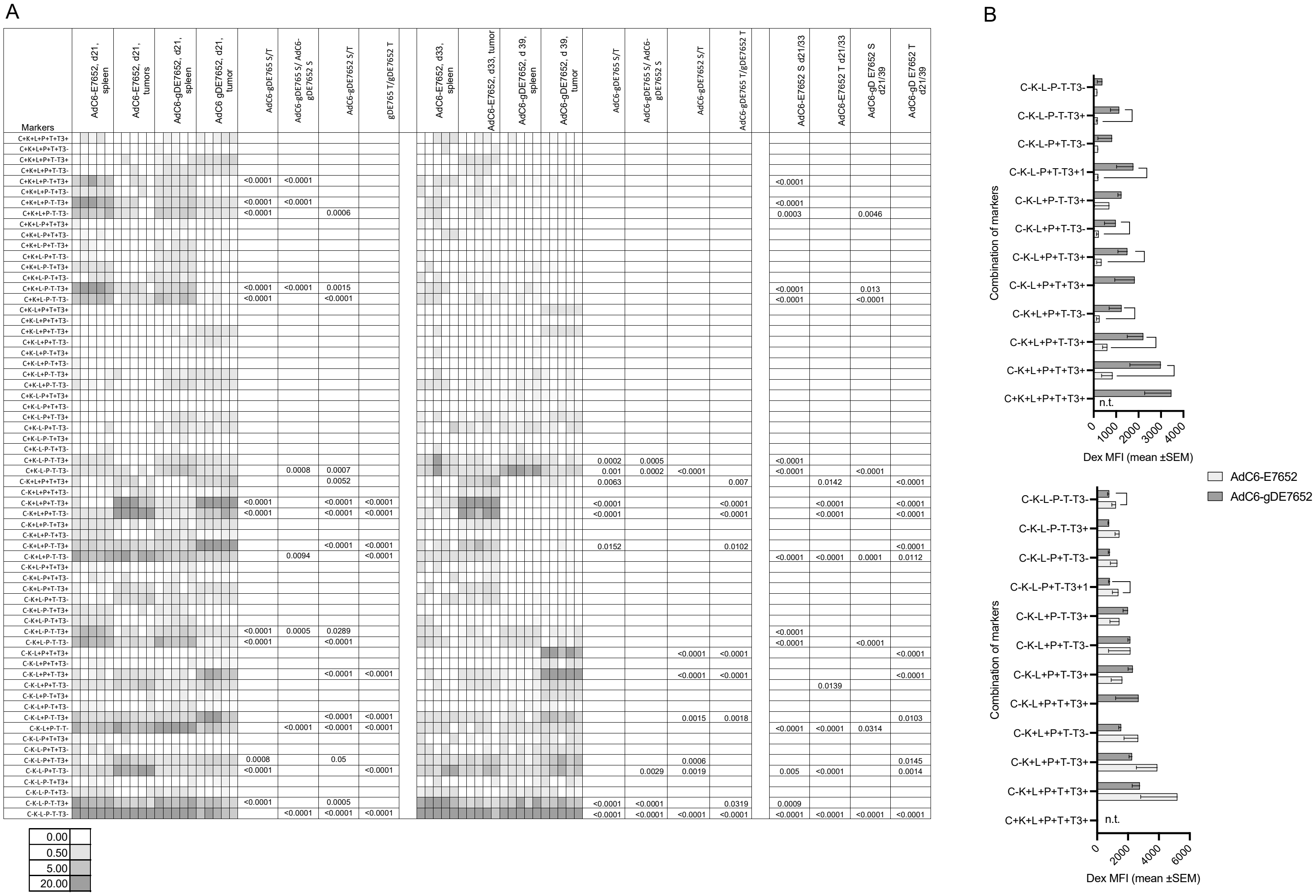

Figure S5. [A] shows as a heatmap percentages of E7-dextramer[+]CD8[+] T cells from spleens and tumors expressing the different combinations of markers. The p-values of significant differences by 2-way Anova are shown to the right. [B] MFI of the E7-dextramer stain of CD8[+] T cells from spleens and tumors expressing the indicated combinations of markers. Differences were calculated by 2-way Anova. S – T cells from spleens, T – T cells from tumors.
